# Risk factors are different for deep and lobar remote hemorrhages after intravenous thrombolysis

**DOI:** 10.1371/journal.pone.0178284

**Published:** 2017-06-22

**Authors:** Luis Prats-Sanchez, Alejandro Martínez-Domeño, Pol Camps-Renom, Raquel Delgado-Mederos, Daniel Guisado-Alonso, Rebeca Marín, Laura Dorado, Salvatore Rudilosso, Alejandra Gómez-González, Francisco Purroy, Manuel Gómez-Choco, David Cánovas, Dolores Cocho, Moises Garces, Sonia Abilleira, Joan Martí-Fàbregas

**Affiliations:** 1 Servei de neuorlogia, Hospital de la Santa Creu i Sant Pau (Biomedical Research Institute, IIB-Sant Pau), Barcelona, Spain; 2 Department of Neurology, Hospital Universitari Germans Trias i Pujol, Badalona, Spain; 3 Department of Neurology, Hospital Clínic de Barcelona, Barcelona, Spain; 4 Department of Neurology, Hospital del Mar, Barcelona, Spain; 5 Department of Neurology, Hospital Universitari Arnau de Vilanova, Lleida, Spain; 6 Department of Neurology, Hospital Moisès Broggi, Sant Joan Despí, Spain; 7 Department of Neurology, Hospital Universitari de Sabadell-Corporació Sanitària Parc Taulí, Sabadell, Spain; 8 Department of Neurology, Hospital General Universitari de Granollers, Granollers, Spain; 9 Department of Neurology, Hospital Verge de la Cinta, Tortosa, Spain; 10 Stroke Programme/Agency for Health Quality and Assessment of Catalonia, Barcelona, Spain; 11 Medicine Department, Universitat Autònoma de Barcelona (UAB), Barcelona, Spain; Medizinische Universitat Innsbruck, AUSTRIA

## Abstract

**Background and purpose:**

Remote parenchymal haemorrhage (rPH) after intravenous thrombolysis is defined as hemorrhages that appear in brain regions without visible ischemic damage, remote from the area of ischemia causing the initial stroke symptom. The pathophysiology of rPH is not clear and may be explained by different underlying mechanisms. We hypothesized that rPH may have different risk factors according to the bleeding location. We report the variables that we found associated with deep and lobar rPH after intravenous thrombolysis.

**Methods:**

This is a descriptive study of patients with ischemic stroke who were treated with intravenous thrombolysis. These patients were included in a multicenter prospective registry. We collected demographic, clinical and radiological data. We evaluated the number and distribution of cerebral microbleeds (CMB) from Magnetic Resonance Imaging. We excluded patients treated endovascularly, patients with parenchymal hemorrhage without concomitant rPH and stroke mimics. We compared the variables from patients with deep or lobar rPH with those with no intracranial hemorrhage.

**Results:**

We studied 934 patients (mean age 73.9±12.6 years) and 52.8% were men. We observed rPH in 34 patients (3.6%); 9 (0.9%) were deep and 25 (2.7%) lobar. No hemorrhage was observed in 900 (96.6%) patients. Deep rPH were associated with hypertensive episodes within first 24 hours after intravenous thrombolysis (77.7% vs 23.3%, p<0.001). Lobar rPH were associated with the presence of CMB (53.8% vs 7.9%, p<0.001), multiple (>1) CMB (30.7% vs 4.4%, p = 0.003), lobar CMB (53.8% vs 3.0%, p<0.001) and severe leukoaraiosis (76.9% vs 42%, p = 0.02).

**Conclusions:**

A high blood pressure within the first 24 hours after intravenous thrombolysis is associated with deep rPH, whereas lobar rPH are associated with imaging markers of amyloid deposition. Thus, our results suggest that deep and lobar rPH after intravenous thrombolysis may have different mechanisms.

## Introduction

The most serious complication of intravenous thrombolysis (IV-rtPA) in patients with acute ischemic stroke is intracerebral hemorrhage (ICH), as these patients have a poor functional outcome and high mortality [1].Thus, it is essential to identify risk factors that signal a future ICH.

Remote parenchymal hemorrhage (rPH) is defined as single or multiple hemorrhages that appear in brain regions without visible ischemic damage detected by cranial computed tomography (CT), remote from the area of ischemic causing the initial symptom [2]. This type of ICH occurs in 1.3–3.7% of patients within 24–36 hours after the infusion of IV-rtPA but risk factors are not well-known [2–4]. It is likely that IV-rtPA triggers rPH when there is an underlying hemorrhagic-prone cerebral angiopathy, such as hypertensive and amyloid angiopathies [2,4,5]. Similarly, spontaneous ICH occurs as the acute manifestation of a chronic cerebral vasculopathy and the most common accepted etiologies are cerebral amyloid angiopathy (CAA) for lobar ICH and hypertension for deep ICH [6]. However, there are no reports of studies that have investigated whether rPH after IV-rtPA risk factors are different depending on its location.

Our aim was to evaluate clinical and radiological risk factors of deep and lobar rPH. We hypothesized that deep rPH is associated with acute or chronic arterial hypertension, whereas rPH in lobar regions is associated with Magnetic Resonance Imaging (MRI) markers of CAA.

## Materials and methods

We report a retrospective, multicenter and descriptive study of patients with ischemic stroke who were treated with IV-rtPA in Catalonia (Spain) between January 2011 and August 2013. Since January 2011 all patient data were prospectively and consecutively included by stroke neurologist in the SONIIA (Sistema Online d'Informació de l'Ictus Agut) registry, a government-mandated registry aiming at continuously monitoring the quality of all reperfusion therapies administered for acute ischemic stroke in Catalonia.

### Ethic statement

All of the authors collected the data used in this study. All data were included in the SONIIA registry, a government-mandated, population-based, externally audited, prospective database that includes all acute ischemic stroke patients treated with reperfusion therapies in the region of Catalonia from January 2011. Written or oral consent was taken from patients on admission about any diagnostic and therapeutic procedure undertaken. The authors also obtained a written or oral consent to use non-personal information included in the SONIIA registry for clinical investigations. Since we applied a retrospective approach, all relevant information was taken from the SONIIA registry, but without re-informing the patients. The SONIIA registry satisfies all legal requirements mandated by the local law regarding protection of personal data. A committee of stroke experts reviewed and ethically approved this study. They also accepted the use of oral consent.

### Study population

We included patients aged ≥ 18 years-old who had a stroke and underwent a follow-up cranial tomography within the first 36 hours of IV-rtPA. ICH complications, including rPH, were classified according to the European Cooperative Acute Stroke Study criteria [7]. Patients with PH without concomitant rPH, stroke mimics and those who underwent endovascular treatment were excluded. We analyzed the results of the MRI only if it was performed within the first 14 days from onset of stroke.

We collected the following variables from 9 different Stroke Centers. Demographics: age (years) and sex; medical history including prior arterial hypertension, diabetes, atrial fibrillation, transient ischemic attack or ischemic stroke; prior medication (use of antiplatelet agents or anticoagulants); stroke severity assessed by the initial National Institutes of Health Stroke Scale (NIHSS); laboratory data (platelet count and coagulation tests including activated partial thromboplastin time and international normalized ratio); onset-to-needle time; hypertensive episodes within the first 24 hours of IV-rtPA infusion defined as an increase more than once of systolic blood pressure ≥185 mmHg or diastolic blood pressure ≥ 105 mmHg within the first 24 hours after IV-rtPA; hyperglycemia if capillary glucose levels were ≥7.7 mmol/L within the first 24 hours after IV-rtPA; type and location of the ICH according to the European Cooperative Acute Stroke Study criteria [7]. Hemorrhagic Infarction (HI) was defined as a petechial infarction without space-occupying effect (HI1: small petechiae; HI2: more confluent petechiae), parenchymal Hemorrhage (PH) was defined as a hemorrhage (coagulum) with mass effect (PH1: <30% of the infarcted area with mild space-occupying effect; PH2: >30% of the infarcted area with significant space-occupying effect), and rPH was defined as an extraischemic remote hematoma, possibly multifocal in nature with and without mass effect (these hemorrhages occurred on CT in brain regions without visible ischemic damage that was remote from an ischemic infarct or in brains without visible ischemic lesion on CT). Lobar rPH were defined as any extra ischemic ICH after IV-rtPA with selective involvement of the cerebral cortex, underlying white matter, or both. Deep rPH were defined as any extra ischemic ICH after IV-rtPA with selective involvement of the basal ganglia, thalamus, internal capsule, external capsule, corpus callosum, deep and periventricular white matter, brainstem or cerebellum. We included patients with concomitant PH and rPH in the rPH group.

The MRI protocol was standardized at each hospital. All studies were performed within the first 14 days from the onset of stroke and were evaluated by one reader (stroke neurologist or neuroradiologist). In most patients, imaging was 1.5-T field strength and always included T1-weighted, T2-weighted, fluid-attenuated inversion recovery (FLAIR), T2*-weighted gradient-recalled echo sequences and diffusion weighted imaging. We evaluated the number and location of cerebral microbleeds (CMB). CMB were defined as small (2-10mm), perivascular, well demarcated hypointense, rounded lesions on MRI on T2*-weighted gradient-recalled echo sequences or sensitive to magnetic susceptibility [8]. The number of CMB was classified as single (n = 1) or multiple (n>1). The location of CMB was classified as deep, lobar or mixed. We classified as deep CMB when they were in the basal ganglia, thalamus, internal capsule, external capsule, corpus callosum, deep and periventricular white matter or brainstem. We classified as lobar CMB when they were in cortical and subcortical regions (including subcortical U fibers). Mixed CMB were considered if the CMB were in deep and lobar locations or CMB in cerebellum. Leukoaraiosis was assessed on FLAIR images with a simplified scale of Fazekas and Schmidt [9]. We defined severe leukoaraiosis as a score of 2 (periventricular smooth halo or deep beginning confluent foci) or 3 (periventricular irregular lesions extending into deep white matter or deep confluent lesions). Cortical superficial siderosis was defined as linear residues of blood products in the superficial layers of the cerebral cortex that showed a characteristic gyriform pattern of low signal on T2* gradient-recalled echo images without corresponding hyperintense signal on T1-weighted or fluid-attenuated inversion recovery images [10].

### Statistical analysis

We compared all of the variables among patients with deep rPH and patients with no ICH, and also among patients with lobar rPH and those with no ICH.

Due to the non-normal distribution of the continuous variables, the bivariate analyses were performed with the Mann-Whitney U-test. Categorical variables were compared with Chi-Square or the Fisher’s exact test. A p value <0.05 was considered statistically significant.

## Results

**Fig 1:**
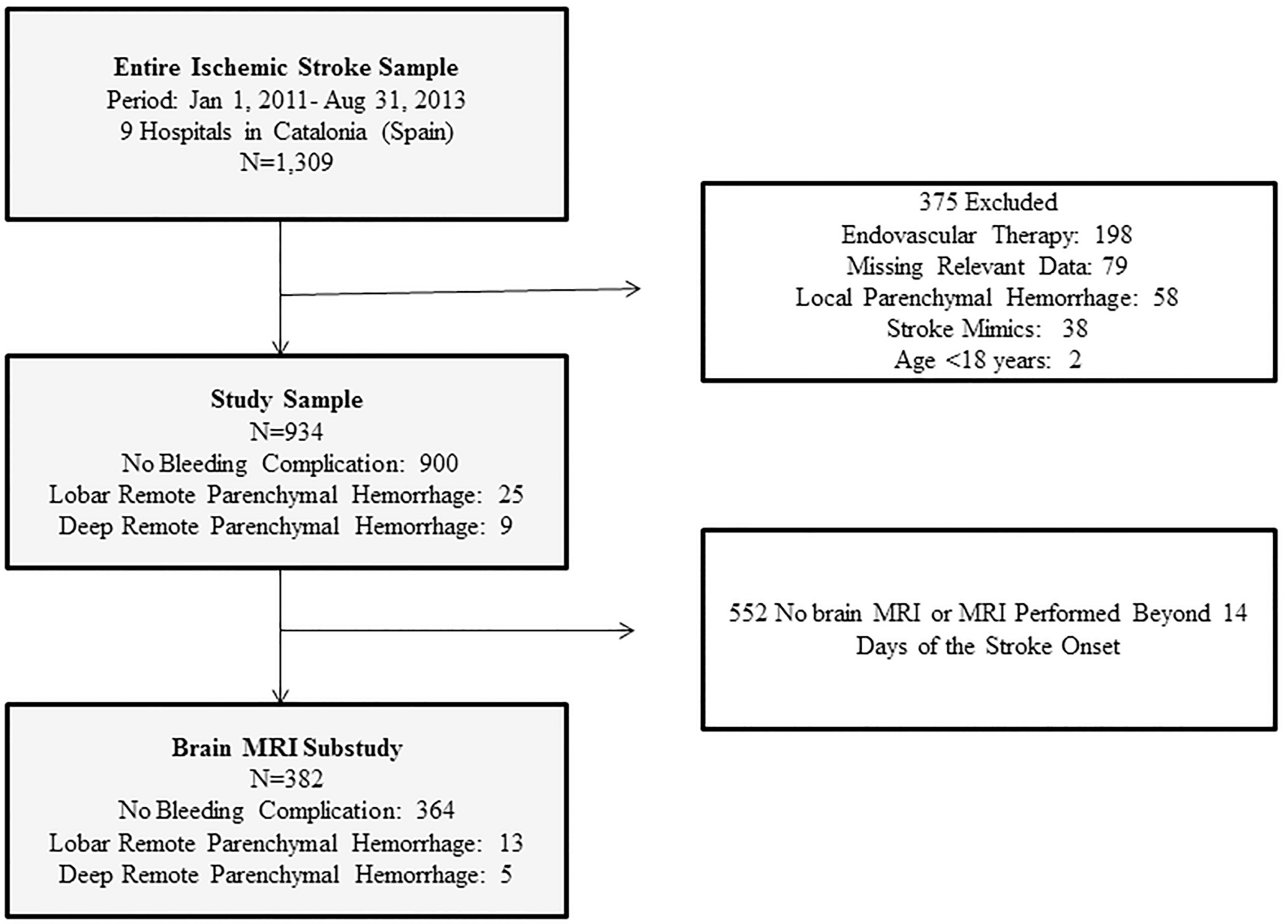
Study flow diagram (patients may had multiple reasons for not being included in the analysis). MRI indicates magnetic resonance imaging.

Of the 1309 patients with stroke treated with IV-rtPA during the study period 378 were excluded; thus 934 patients were included in the final analysis (Fig 1). Their mean age was 73.9±12.6 years and 52.8% were men. We observed 25 (2.6%) patients with a lobar rPH, 9 (0.9%) with a deep rPH and 900 with no ICH. We evaluated MRI results of 382 patients: 13 with lobar rPH, 5 with deep rPH and 364 with no ICH.

Demographic variables, medical history, stroke severity, blood tests and onset-to-needle time were not different between patients with deep rPH or lobar rPH and patients without any ICH. (Table 1**)**

**Table 1:** Bivariate analyses of baseline variables of deep rPH, lobar rPH and patients without ICH.

	Deep rPH n = 9	Lobar rPH n = 25	No ICH n = 900	p value Deep rPH vs No ICH	p value Lobar rPH vs No ICH
Age, years, median (IQR)	78 (73–85)	78 (72–84)	77 (67–83)	0.46*	0.39*
Men, n (%)	3/9 (33.3)	13/25 (52)	477/900 (53)	0.32†	0.99†
Hypertension, n (%)	5/9 (55.5)	17/25 (68)	651/900 (72.3)	0.27†	0.65†
Diabetes, n (%)	3/9 (33.3)	4/25 (16)	213/900 (23.6)	0.45†	0.47†
Atrial fibrillation, n (%)	3/9 (33.3)	6/25 (24)	213/900 (23.6)	0.45†	0.99†
TIA/Stroke, n (%)	2/9 (22.2)	4/25 (16)	130/900 (14.4)	0.62†	0.77†
Antiplatelets, n (%)	4/9 (44.4)	13/25 (52)	330/900 (36.6)	0.73†	0.14†
Anticoagulants, n (%)	2/9 (22.2)	1/25 (4)	60/900 (6.6)	0.12‡	0.99‡
Baseline NIHSS, median (IQR)	12 (6–15.5)	11 (5–19)	10 (6–17)	0.88*	0.66*
Onset-to-needle time (min), median (IQR)	150 (125–257)	144 (97–187)	135 (99–180)	0.08*	0.53*
Platelet count, 10^9^/L, median (IQR)	210 (162–236)	208 (180–243)	215 (177–257)	0.69*	0.43*
aPTT (ratio), median (IQR)	0.9 (0.8–0.9)	0.92 (0.85–0.94)	0.89 (0.83–0.94)	0.70*	0.44*
INR, median (IQR)	1.0 (1.0–1.2)	1.0 (0.9–1.1)	1.0 (0.9–1.1)	0.42*	0.92*
INR anticoagulants, median (IQR)	1.4	1.3	1.3 (1.2–1.5)	0.76*	0.95*
Hypertension 24h, n (%)	7/9 (77.7)	6/23 (26)	206/884 (23.3)	<0.001†	0.60†
Hyperglycemia 24h, n (%)	2/9 (22.2)	3/23 (13)	180/789 (22.8)	0.99†	0.15†
1 CMB, n (%)	1/5 (20)	7/13 (53.8)	29/364 (7.9)	0.34‡	<0.001‡
>1 CMB, n (%)	0/5 (0)	4/13 (30.7)	16/359 (4.4)	0.99‡	0.003‡
Lobar CMB, n (%)	1/5 (20)	7/13 (53.8)	11/364 (3.0)	0.15‡	<0.001‡
Deep CMB, n (%)	0/5 (0)	0/13 (0)	5/364 (1.3)	0.99‡	0.99‡
Mixed CMB, n (%)	0/5 (0)	0/13 (0)	9/364 (2.4)	0.99‡	0.99‡
Severe Leukoaraiosis, n (%)	0/5 (0)	10/13 (76.9)	153/360 (42)	0.07‡	0.02‡
CSS, n (%)	1/5 (20)	1/13 (7.6)	4/354 (1.1)	0.06‡	0.16‡

Values are expressed as absolute counts and percentage for categorical variables and median and interquartile range (IQR) for continuous variables. P values are given for difference between patients with deep or lobar remote parenchymal hemorrhage (rPH) and patients without any intracranial hemorrhage (ICH). The p values were obtained by Mann-Whitney *U* Test (*), χ2 Test (†) or Fisher exact test (‡). TIA, indicates transient ischemic attack; NIHSS, National Institutes of Health Stroke Scale; aPTT, activated partial thromboplastin time; INR, international normalized ratio; INR anticoagulants, international normalized ratio in patients who were treated with oral anticoagulants; Hypertension 24h, hypertensive episodes within first 24 hours of intravenous thrombolysis; Hyperglycemia 24h, capillary glucose ≥7.7 mmol/L within the first 24 hours of intravenous thrombolysis; CMB cerebral microbleeds; Severe Leukoaraiosis, a score of 2 or 3 in a simplified scale of Fazekas and Schmidt [9]; CSS, cortical superficial siderosis.

Hypertensive episodes within the first 24 hours of IV-rtPA were significantly more common in patients with deep rPH compared to patients with no ICH (77.7% vs 23.3%, p<0.001). The presence of CMB (53.8% vs 7.9%, p<0.001), lobar distribution of CMB (53.8% vs 3.0%, p<0.001), multiple CMB (30.7% vs 4.4%, p = 0.003) and severe leukoaraiosis (76.9% vs 42%, p = 0.02) were significantly more frequent in patients with lobar rPH compared to those without ICH. (Table 1).

## Discussion

We found that deep rPH and lobar rPH exhibited different risk factors. Deep rPH was associated with hypertensive episodes within the first 24 hours after IV-rtPA infusion, whereas lobar rPH was associated with neuroimaging surrogate markers of CAA.

There is increasing evidence of a link between CAA and ICH after IV-rtPA [2,11,12]. A large meta-analysis [11] of patients with ischemic stroke who were treated with IV-rtPA showed that patients who exhibited CMB and had a high burden of CMB were associated with a high risk symptomatic ICH. A positron emission tomography study [12] found an association between patients with CAA and IV-rtPA associated ICH. However, in these studies [11,12] a separate analysis of rPH was not reported. Recently, a multicenter study [2] showed that lobar CMB were associated with rPH. In addition, rPH after IV-rtPA has been documented to occur at a site of CMB [13]. Leukoaraiosis or white matter hyperintensities include a ubiquitous phenomenon of ageing, but occur with much greater volume in CAA patients [14]. Progressive leukoaraiosis has been associated with incident lobar hemorrhages in CAA patients [15]. In addition, a previous study showed that severe leukoaraiosis increased the risk of rPH [4]. Finally, a pathological study of 5 patients with ICH after thrombolytic therapy in myocardial infarction, showed that 3 of them had CAA [16]. All these findings, although preliminary, suggest that a proportion of rPH is explained by the vascular fragility of CAA together with the toxicity of IV-rtPA.

In patients without hypertension and a deep or infratentorial spontaneous ICH, blood pressure ≥160/100mmHg is sufficient evidence to conclude that bleedings are hypertension-related [17]. Thus, in patients with an acute ischemic stroke, we feel safe in speculating that deep rPH is the result of two concomitant processes (hypertensive episodes and IV-rtPA). Similarly, patients with hypertension who are under treatment with anticoagulants have a high risk of a spontaneous deep ICH [6]. Consistent with our findings, two large studies [3,18] showed that a high systolic blood pressure after IV-rtPA is associated with rPH and any symptomatic ICH. We do not know whether deep rPH can be prevented by a rapid and persistent control of blood pressure in the acute phase of stroke, but this question may be answered by the results of the on-going ENCHANTED trial [19].

Our results must be interpreted cautiously. First, this is a retrospective analysis from a prospectively collected database. Like any register, reporting bias cannot be totally excluded, although it was a mandatory and externally audited registry. Second, MRI results were evaluated by different investigators according to local protocols, and without intra or inter-rater variability analyses. Third, all of the MRI studies were performed after IV-rtPA. It is known that 4.9–12.7% of acute stroke patients treated with IV-rtPA develop new CMB within the first week after symptom onset, and thus, not all CMB demonstrated in the MRI may be chronic in nature [20,21]. In addition, a pooled meta-analysis showed that patients with new CMB after IV-rtPA have an increased risk of rPH [22]. Fourth, we categorized hypertensive episodes within the first 24 hours after IV-rtPA, but we did not know the number of hypertensive episodes of the exact time that blood pressure was uncontrolled. Fifth, our registry did not include the total dose of IV-rtPA and the fibrinogen levels. It is documented that some patients with ischemic stroke or myocardial infarction after a high dose of IV-rtPA have higher fibrinogen degradation products and lower fibrinogen levels [16,23]. Fibrinogen degradation coagulopathy after IV-rtPA is a relevant cause of symptomatic ICH and other major systemic bleeding events [16,23]. Sixth, the low number of events was a limitation to find other etiologies of rPH such as the use of anticoagulants, blood dyscrasias or structural vascular abnormalities [7,24]. A meta-analysis suggested an increased risk of symptomatic ICH after IV-rtPA in patients with subtherapeutic (international normalized ratio≤1.7) warfarin therapy but a detailed analysis about rPH was not provided [24]. In our study there were 63 patients (2 deep rPH, 1 lobar and 60 without any ICH) who were treated with oral anticoagulants prior to IV-rtPA but the international normalized ratio was not different between groups. Finally, we studied a small number of patients with rPH. Thus, the results of the statistical analysis should be interpreted cautiously, especially regarding the MRI substudy. In addition, due to the low number of events, we could not perform multivariate analyses to adjust for confounding factors.

Although our findings are exploratory, they may facilitate the design of future studies to clarify the risk factors of deep and lobar rPH. We believe that our results contribute to the growing evidence of the association among patients with a high MRI burden of small-vessel disease and rPH. However, our data do not justify withholding IV-rtPA in patients who are otherwise candidates for this treatment or for primary endovascular reperfusion therapy. Additionally, it seems prudent to prevent hypertensive episodes above the limits established by current guidelines.
